# Barriers and Facilitators When Implementing Web-Based Disease Monitoring and Management as a Substitution for Regular Outpatient Care in Pediatric Asthma: Qualitative Survey Study

**DOI:** 10.2196/jmir.9245

**Published:** 2018-10-30

**Authors:** Lara S van den Wijngaart, Wytske W Geense, Annemie LM Boehmer, Marianne L Brouwer, Cindy AC Hugen, Bart E van Ewijk, Marie-José Koenen-Jacobs, Anneke M Landstra, Laetitia EM Niers, Lonneke van Onzenoort-Bokken, Mark D Ottink, Eleonora RVM Rikkers-Mutsaerts, Iris Groothuis, Anja A Vaessen-Verberne, Jolt Roukema, Peter JFM Merkus

**Affiliations:** 1 Department of Pediatric Pulmonology, Amalia Children's Hospital Radboud Institute of Health Sciences Radboud University Medical Center Nijmegen Netherlands; 2 IQ Healthcare Radboud Institute of Health Sciences Radboud University Medical Centre Nijmegen Netherlands; 3 Department of Pediatrics Maasstad Hospital Rotterdam Netherlands; 4 Department of Pediatrics Canisius Wilhelmina Hospital Nijmegen Netherlands; 5 Department of Pediatrics Tergooi Hospital Blaricum Netherlands; 6 Department of Pediatrics Tergooi Hospital Hilversum Netherlands; 7 Department of Pediatrics Maas Hospital Pantein Boxmeer Netherlands; 8 Department of Pediatrics Rijnstate Hospital Arnhem Netherlands; 9 Department of Pediatrics Maxima Medical Center Veldhoven Netherlands; 10 Department of Pediatrics Medical Spectrum Twente Hospital Enschede Netherlands; 11 Department of Pediatric Pulmonology Leiden University Medical Center Leiden Netherlands; 12 Department of Pediatric Pulmonology Juliana Children's Hospital Haga Hospital The Hague Netherlands; 13 Department of Pediatrics Amphia Hospital Breda Netherlands

**Keywords:** asthma, barriers and facilitators, eHealth, pediatric, Web-based monitoring

## Abstract

**Background:**

Despite their potential benefits, many electronic health (eHealth) innovations evaluated in major studies fail to integrate into organizational routines, and the implementation of these innovations remains problematic.

**Objective:**

The purpose of this study was to describe health care professionals’ self-identified perceived barriers and facilitators for the implementation of a Web-based portal to monitor asthmatic children as a substitution for routine outpatient care. Also, we assessed patients’ (or their parents) satisfaction with this eHealth innovation.

**Methods:**

Between April and November 2015, we recruited 76 health care professionals (from 14 hospitals). During a period of 6 months, participants received 3 questionnaires to identify factors that facilitated or impeded the use of this eHealth innovation. Questionnaires for patients (or parents) were completed after the 6-month virtual asthma clinic (VAC) implementation period.

**Results:**

Major perceived barriers included concerns about the lack of structural financial reimbursement for Web-based monitoring, lack of integration of this eHealth innovation with electronic medical records, the burden of Web-based portal use on clinician workload, and altered patient-professional relationship (due to fewer face-to-face contacts). Major perceived facilitators included enthusiastic and active initiators, a positive attitude of professionals toward eHealth, the possibility to tailor care to individual patients (“personalized eHealth”), easily deliverable care according to current guidelines using the VAC, and long-term profit and efficiency.

**Conclusions:**

The implementation of Web-based disease monitoring and management in children is complex and dynamic and is influenced by multiple factors at the levels of the innovation itself, individual professionals, patients, social context, organizational context, and economic and political context. Understanding and defining the barriers and facilitators that influence the context is crucial for the successful implementation and sustainability of eHealth innovations.

## Introduction

In the last decades, the use of information and communication technology (ICT) in health care, also known as electronic health (eHealth), has rapidly evolved. It is widely used as a tool for improving health care, it delivers health care to geographically remote areas, it has the potential to reduce costs, and it improves health-related behavior and long-term management of chronic diseases. [1-3] Despite their potential benefits, many eHealth innovations evaluated in major studies do not reach the stage of structural implementation in daily practice and policy [4,5]. In The Netherlands, eHealth has no place yet in pediatric asthma management [6].

The barriers and facilitators for successful implementation have been the topic of several review papers [7-10], and Granja et al [7] suggested to also consider the scientific assessment of the quality of care and of the financial consequences of eHealth to optimize chances of successful implementation. We studied the implementation of eHealth prospectively, using our Web-based asthma monitoring management as an example.

In 2011, a Web-based portal to monitor asthmatic children was developed with the aim to change current pediatric asthma care and to (partly) substitute routine outpatient visits by Web-based monitoring. This Web-based portal, also called the virtual asthma clinic (VAC) for children, consisted of an information module, a forum for peers, a communication module, and an individual treatment plan for patients. Initially, we focused on providing evidence for a positive impact of this eHealth innovation on clinical endpoints. After 16 months of follow-up, the number of symptom-free days and the degree of asthma control improved in children who received care using the VAC, and we concluded that routine outpatient visits could partly be replaced by monitoring asthmatic children using eHealth. [11] Furthermore, the outcome of the cost-effectiveness analysis was in favor of using Web-based monitoring as a substitution for outpatient visits from a health economics perspective [12].

With this evidence of the impact of the VAC on clinical outcomes, the logical next step in the organizational process was the effective implementation of the VAC in a larger number of Dutch hospitals in order to become an integral part of current pediatric asthma management.

The main factor that complicates implementation is either human or organizational [13-16]. Often, the consequence is that eHealth is no longer supported when the funding supporting the study ends [17]. Additionally, future dissemination and implementation of the innovation are almost always neglected during its developmental stage, resulting in the poor sustainability of the innovation [17,18]. Thus, it is important to acquire a good understanding of the problem, the target group, its setting, the obstacles to change or resolve, and to “start with the end in mind.” The first step in an implementation process should be to recognize, identify, and understand barriers and facilitators, crucial for addressing blockages to implementation and inventing strategies to improve the effective use and sustainability of eHealth in daily practice [19,20]. To examine barriers and facilitators, the model of Grol and Wensing can be used, proposing that barriers and facilitators can be examined at 6 different levels: innovation, individual professional, patient, social context, organizational context, and economic and political context (see Multimedia Appendix 1) [20].

Based on this model, we conducted a qualitative survey study with the aim to identify barriers and facilitators experienced by Dutch health care professionals and patients (or their parents) when implementing eHealth in routine pediatric asthma care. In addition, we summarize our lessons learned in recommendations that we consider relevant for successful implementation and sustainability of eHealth in general.

## Methods

### Study Design

This qualitative survey study utilized data collected from health care professionals including pediatricians, pediatric pulmonologists, nurse practitioners, pediatric (respiratory) nurses, and children or their parents [21]. We conducted the study in 14 hospitals (11 general and 3 tertiary, both urban and rural) in The Netherlands between April and December 2015.

### Participants

The 8 hospitals originally participating in the randomized controlled trial (RCT) [11] were approached to also collaborate in this study. Of these, 6 hospitals agreed to participate, and 2 hospitals declined for logistic reasons. The other hospitals (8 in total) were contacted by email. All hospitals agreed to start with the implementation of the VAC. Finally, a total of 14 hospitals intended to participate in this study. In each hospital, several health care professionals confirmed their willingness to participate including pediatric pulmonologists, pediatricians, pediatric residents, nurse practitioners, or pediatric respiratory nurses. The number of participating health care professionals differed between hospitals. Also, patients (or their parents) were asked to complete a questionnaire about their satisfaction with the VAC after 6 months; they only completed 1 questionnaire at the end of the implementation (at 6 months).

### Innovation

The VAC for children is a Web-based portal for children with asthma. It consists of a general information module and a secure private section where children (or their parents) can log in to communicate easily with their asthma team, download or consult their individual treatment plan, and complete a validated questionnaire for Web-based monitoring of their asthma control. Multimedia Appendix 2 shows different screenshots of the innovation (the individual care plan of a dummy patient).

Implementation of the VAC in pediatric asthma care included the use of the VAC in daily practice without a fixed protocol for the use of this innovation. Despite the existence of a national guideline for pediatric asthma management in The Netherlands, subtle differences between hospitals and health care professionals may exist (eg, provided information about asthma, the frequency of follow-up, preferences of prescribed medication). In addition, use of the VAC was tailored to local (organizational) preferences, for example, whether a nurse practitioner was involved in the use of the VAC or which professional was primarily responsible for contact with the patient. No restrictions were made about the frequency of outpatient visits or Web-based monitoring per patient.

Before implementation of the VAC, a 2-hour introductory visit was planned in each hospital, accessible to all participating professionals. These introductory visits were given by the same person (LvdW) and were aimed at adequately informing professionals about the purpose and details of the study and practical use of the VAC. The information provided was semistructured in each hospital. This visit included a real-time demonstration of the VAC and the different functions within the innovation. Further, questions by professionals were answered, and contact information and user manuals for patients and professionals were provided. Patients (or their parents) were informed about the use of the VAC by their asthma team and also received a written instruction manual. During the implementation process, all professionals could contact the help desk in case of existing problems.

Successful implementation was defined as using the VAC on a regular basis in daily pediatric asthma care, patients using the VAC regularly, and a positive attitude (of all end-users) toward continuing the use of the VAC in the future.

### Data Collection and Analysis

Information about barriers and facilitators for successful implementation were collected by structured Web-based questionnaires (closed survey), which were completed by all professionals at the start of implementation and after 3 and 6 months using the same questionnaire at these different time points. Participation was voluntary, and health care professionals and patients could withdraw from the study at any time for any reason. No incentives were offered. Participants had 1 month to complete the questionnaire before the survey was closed.

Questionnaires were based on the model of Grol and Wensing, proposing that barriers and facilitators could be examined at 6 different levels: innovation, individual professional, patient, social context, organizational context, and economic and political context [20]. Questionnaires consisted of both open and multiple choice questions (see Multimedia Appendix 3). Participants received an email with a link to the questionnaire (email survey). No pilot-testing was done before distribution of these questionnaires, but the usability and technical functionality of the electronic questionnaire had been tested by the research team before fielding the questionnaire. All responses were automatically captured, analyzed, and anonymized.

In case of missing questionnaires, an automatically generated reminder was sent after 1 and 2 weeks. When a professional did not complete the questionnaire after 2 reminders, the link to the questionnaire was closed, and the questionnaire was considered missing. Only completed questionnaires were analyzed (as it was not possible to close the survey when items were missing).

Barriers and facilitators for implementation of the VAC in daily practice were identified from the open-ended questions and assessed using qualitative analysis. Barriers and facilitators were divided into different categories and organized into themes using the adapted model for understanding change at different levels of health care [20]. Two authors (LvdW and WG) identified and categorized the facilitators and barriers independently, based on the answers to the open-ended questions. In cases of disagreement, identification and categorization were discussed to reach consensus.

Descriptive statistics were used to analyze the multiple choice questions (Likert scale responses).

After 6 months, health care professionals were asked to send a questionnaire on patient satisfaction to their patients (or parents) via the VAC. The researchers had no influence on how many patients were reached because they had no access to local clinical data for medicolegal reasons. Therefore, no information was provided about the severity of their asthma and current medication or treatment regimes. Questions were addressed pertaining to the different modules of the VAC (Multimedia Appendix 4), the innovation, the (Children) Asthma Control Test questionnaire for Web-based monitoring disease deterioration, the communication module, the individual treatment plan, privacy and security, and overall satisfaction. Participants completed the questionnaire on a voluntary basis. Results of this questionnaire were used in this study and to update and further develop the VAC according to users’ needs and wishes.

### Ethics

The study was approved by the local medical ethics committee (Commisie Mensgebonden Onderzoek Nijmegen-Arnhem), which waived written informed consent.

## Results

### General Results

#### Health Care Professionals

The characteristics of all participating hospitals, including the number of participating health care professionals and whether implementation was successful or not, can be found in Multimedia Appendix 5.

Characteristics of the participating professionals, covering a wide range with respect to age and experience, are shown in Table 1. Initially, a total of 75 professionals intended to participate. The first questionnaire at the beginning of the study was completed by 68% (51/75) professionals: pediatric pulmonologists (14/51, 27%), pediatricians (19/51, 37%), nurse practitioners (7/51, 14%), and pediatric (respiratory) nurses (11/51, 22%). The response rates of the professionals at the beginning of the implementation and after 3 and 6 months were 67% (51/76), 63% (48/76), and 51% (39/76), respectively.

The mean age of all professionals was 47 (SD 8) years, and the mean work experience was 14 (SD 8) years. The majority of the professionals (37/51, 73%) had no experience with eHealth in daily practice at the start of the study.

Almost 70% (35/51) of the professionals were present at the introductory visit and received detailed information about the study and the use of the VAC. The other professionals were informed about the VAC by colleagues. Only 2% (1/51) did not receive any information before starting to use the VAC. All participants concluded that information provided at the introductory visits and the instruction manual were clear. Only 18% (9/51) of the professionals read the instruction manual thoroughly.

Implementation of the VAC was unsuccessful in 29% (4/14) hospitals. In 1 hospital, this was due to insufficient staff; just 1 pediatric pulmonologist was available for the implementation of the VAC, and for this physician, the time investment was too much in addition to his regular work. No specific reasons for unsuccessful implementation in the other 3 hospitals could be assessed due to missing questionnaires at follow-up. No further follow-up was done with the hospital representatives who initially agreed to participate.

#### Characteristics of Patients and Their Parents

At the end of the study, 66 parents completed the voluntary questionnaire to provide information about the VAC. Most of their children were male (51/66, 77%) with a mean age of 10.1 (SD 2.5) years. On average, these parents used the VAC for 7.6 (SD 4.7) months. There were 10 children with asthma who completed the questionnaire.

### Barriers and Facilitators

Barriers and facilitators were divided into 6 categories and organized into themes using the adapted model for understanding change at different levels of health care [20]. Table 2 provides an overview of the framework with barriers and facilitators in each theme.

#### Innovation

By *innovation*, we mean “the eHealth innovation (or the object) of the implementation process,” in this study, the VAC. There were 4 categories related to this theme: (1) attractiveness; (2) amount of information; (3) (dis)advantage; and (4) accessibility and usability.

Several professionals stated the *attractiveness* of the innovation as a facilitator, as the innovation was clear, easy, and user-friendly. Also, the innovation provided bundled, reliable, and age-adjusted *information* about asthma for children and their parents.

Professionals also mentioned the *advantage* of the VAC to substitute routine outpatient visits as a major facilitator. However, other professionals defined restrictions in the use of the innovation. For example, their assumption or cognition was that Web-based monitoring only based on the asthma control test, which is sensitive to confounding factors, is not an adequate substitution for these visits. They also stated that the missing integration between the VAC and electronic medical records (EMRs) was a barrier for the use of the VAC in daily practice, as this resulted in an extra workload to keep both systems up-to-date.

Interactive educational methods (ie, demonstration and instruction of the VAC at the beginning of the study), enthusiasm, and motivation of those responsible for the innovation were considered important contributors to the *accessibility* of the innovation. The accessibility of the innovation was also facilitated by the available user manual and (technical) support service during the study. Both items were central facilitators in helping professionals to use the eHealth innovation in daily practice. Other professionals had some concerns about the privacy of patient data when using eHealth and concerns about the *usability* of the innovation when ICT problems occurred.

**Table 1:** Characteristics of participating health care professionals.

Characteristics	Pediatric pulmonologist (n=14)	Pediatrician (n=19)	Nurse practitioner (n=7)	Pediatric (respiratory) nurse (n=11)
Age (years), mean (SD)	47 (8.5)	46 (9.6)	50 (5.1)	48 (5.7)
Gender (male), n (%)	7 (50)	9 (47)	0 (0)	0 (0)
Work experience (years), mean (SD)	10 (6.5)	12 (8.0)	18 (9.1)	18 (6.0)
Present at introductory visit, n (%)	9 (64)	13 (68)	5 (71)	8 (73)

**Table 2:** Themes, categories, and facilitators and barriers.

Themes and category		Facilitators	Barriers
**Innovation**			
	Attractiveness	User-friendliness of the program	Usage restriction of the innovation
	Amount of information	Bundled, reliable, and age-adjusted information about asthma	Asthma control test is sensitive but not specific for poor asthma control (eg, a common cold may falsely suggest poor asthma control)
	(Dis)advantage	Possibility of frequent monitoring patients’ symptoms	Link with patients’ electronic health records is missing
	Accessibility and usability	Possibility to ask questions (helpdesk) User manual with instruction at start	The (theoretical) possibility that privacy of patients’ data could not be fully guaranteed Usability in case of ICT^a^ problems
**Individual professionals**			
	Attitude of professionals	Attitude of the health care professional (believe in eHealth, convinced of the value of the innovation in daily practice)	Attitude of the health care professional (not convinced of the value of the innovation in daily practice)
	Professional skills	Enrichment of work	No substitution for face-to-face contact
	Knowledge and awareness of eHealth	Expectation that parents and children favor the innovation Accessible contact and improvements in relationship with patients More time for complex patients Customizing care to the individual patient Possibility to gain experience with eHealth Results of the randomized controlled trial about effectiveness of the innovation were positive Experience with the use of the innovation in an earlier study	Risk of losing patients out of sight Extra way of communication Adequate and timely response to messages is difficult to ensure Less patient contact can have a negative effect on the professional’s own development Difficulty to motivate colleagues to use the innovation Lack of time to explore the innovation Management imposed the innovation Difficulty to recruit patients No routine use of the innovation Lack of knowledge or (computer) skills
**Patients**			
	(Dis)advantage for the patient	Fewer outpatient visits	Less contact with the health care professional
	Patient satisfaction and compliance	Less absenteeism from school	More (daily) confrontation with the diagnosis of asthma
	Accessibility and usability	Promoting patients’ compliance, self-management, and knowledge Patient satisfaction Less focus on illness of the child Improvement of security and privacy with the use of the innovation	The innovation is not applicable for all patients Ensure continuous use of the innovation by patients Lack of access to the internet Different options in the innovation Patients were inadequately instructed about the innovation
**Social context**			
	(Lack of) sufficient interprofessional collaboration	Better collaboration between health care professionals	Unclear allocation of tasks between health care professionals
	Substitution of tasks or care by health care professionals	Substitution of care by health care professionals	—^b^
	Care according to current guidelines	Possibility to give care according to the most recent guidelines	—^b^
**Organizational context**			
	Organization of care or care processes	Care logistics is better organized nowadays	Implementation of the innovation in daily practice was unclear or incomplete
	Time	Fewer outpatient visits resulting in more time	Link to patients’ electronic health dossiers is missing
	ICT infrastructure	Time saving and efficient Smaller workload for personnel Positive public relations for department and hospital	Lack of involvement of management during implementation Lack of promotional material (ie, leaflets) Extra workload, time, and administration Delay at start-up ICT problems
**Economic and political context**			
	Financial arrangements	Complementary to current asthma management Keep up with current developments Improvement of care	Uncertainty of future of pediatricians Similar eHealth innovations had no added values Lack of reimbursement for Web-based monitoring

^a^ICT: information and communication technology.

^b^Barriers were not described in this theme and/or category.

#### Individual Professionals

There were 3 categories defined: (1) knowledge and awareness of eHealth; (2) attitude of health care professionals toward the innovation; and (3) professional skills.

At the start of the study, 20% (10/51) of the professionals concluded they had insufficient *knowledge* about the VAC and for whom it was intended. Almost 40% (20/51) did not know what was expected with regard to working with the VAC, and 8% (4/51) had doubts about its use in daily practice.

The professionals’ commitment toward eHealth appeared to be of great importance for the successful implementation of an eHealth innovation. A *positive attitude* toward the use of eHealth seemed the most important facilitator for easy and quick implementation of the VAC in daily practice. By using the VAC, professionals experienced eHealth as an enrichment of their work. They were more able to customize care to the individual patient, and this “personalized eHealth” matches the needs and wishes of both professionals and patients. Besides this, the use of the VAC was reported to facilitate the provision of asthma care according to current guidelines. Also, they emphasized the accessible contact, the improvement of the patient-doctor relationship, and the fact that they had more time for complex patients.

In contrast, when professionals *were not convinced* of the added value of the eHealth innovation, successful implementation of the innovation was unlikely despite all efforts. As reasons, professionals mentioned the risk of losing sight of patients and the fact that Web-based monitoring could never substitute face-to-face contacts due to, for example, the missing interpersonal relationship. Additionally, other professionals saw the VAC as a possible barrier for professionals’ own development due to fewer patient contacts.

Nevertheless, most professionals had a positive attitude about eHealth overall (*“it is part of health care”*). They stated that with the use of the VAC they had a great opportunity to start using eHealth in daily practice in a structured, solid, and easy way.

*Professional skills* and the cognition of professionals toward these skills were also important. Some professionals experienced a lack of (eHealth, ICT, or computer) knowledge or a lack of (computer) skills. Further, they stated that there was no time to explore the VAC adequately and improve their skills. The majority of the professionals mentioned that they wanted to spend more time using the VAC to continue to improve these skills in order to take full advantage of all the various features of the innovation. However, they lacked time due to their work and the responsibilities of taking care of patients.

#### Patient Level

At the level of the patient, professionals mentioned several facilitators and barriers for the implementation of the VAC, which were divided into 3 categories: (1) (dis)advantage for the patient; (2) patient satisfaction and compliance; and (3) accessibility and usability.

The *main advantages* reported by patients were fewer routine outpatient visits and, as a result of this, less absence from school and work (parent-related). Professionals experienced high *patient satisfaction*. Further, they stated that *patients’ compliance*, self-management, and knowledge were promoted by the use of the VAC. With the use of the VAC, patients can easily and proactively participate in the treatment of their own disease, and asthma management is more based on shared decision making. Also, Web-based monitoring resulted in less focus on the illness of the child.

Notably, for other professionals, this last argument was not a facilitator but a barrier. They mentioned that Web-based monitoring emphasized the confrontation with asthma, as a result of the monthly questionnaire. The fact that the VAC is not suitable for all patients was found to be another barrier to implementation according to professionals. For example, patients with poor perception of their asthma symptoms were not considered suitable for Web-based monitoring as a substitution for routine outpatient visits. Therefore, they emphasized that less contact with professionals is not always feasible or wise. A potential risk of Web-based monitoring via the VAC is the patient compliance: how can professionals ensure continuous use of the innovation by patients? Compliance may be influenced by patients’ motivation, symptom burden, and cognitive abilities. With a patient’s lack of motivation for completing the assessment tool, Web-based monitoring as a substitution for outpatient visits is impossible and will not result in proper asthma care. Another potential barrier mentioned by professionals was the concern about a lack of access to the internet for some patients, resulting in selection bias and the innovation not being *accessible* for all patients. However, in The Netherlands, over 96% of the Dutch households had access to the internet in 2016.

The majority (56/60, 93.3%) of the parents concluded that the VAC had added value to current daily pediatric asthma management. Parents were satisfied with the attractiveness, usefulness, and user-friendliness of the innovation. Further, the C-ACT questionnaire was found to be an adequate tool for Web-based monitoring of disease deterioration. Because of the frequent follow-up by C-ACT, most parents were more aware of their child’s asthma symptoms. They concluded that this eventually led to better asthma control. The perceived threshold to communicate through the VAC was low, communication was easy, and they concluded that the asthma team responded within the agreed timeframe (of 2 working days) most of the time. The majority of the parents experienced more control of the asthma of their child, felt to be more independent, and experienced better cooperation with the physician by using the VAC. Security was no issue for 96% (58/60) of the patients or parents, as they trusted that personal information on the VAC was safe. Only 10% (6/60) of the parents missed out on the personal or face-to-face contact with professionals. Overall, the majority of the parents (56/60, 93%) concluded that the VAC had additional value to current daily pediatric asthma management and wanted to use the VAC in the future. The VAC was scored an 8 or higher (based on a 10-point scale, with 10 corresponding to maximum satisfaction) by 78% (47/60) of the parents. Similar scores were reported by the children completing the questionnaire.

#### Social Context

There were 3 categories defined at the level of social context: (1) (lack of) sufficient interprofessional collaboration; (2) substitution of tasks or care by health care professionals; and (3) care according to current guidelines.

First, regarding *sufficient interprofessional collaboration,* most professionals stated that the use of an eHealth innovation like the VAC improved the collaboration between professionals. However, this was only the case when all participating professionals adjusted their tasks before starting to use the VAC. When the asthma team did not adjust or coordinate tasks accordingly, professionals experienced a lack of sufficient interprofessional collaboration resulting in inadequate use of the eHealth innovation.

Second, the use of the VAC facilitated *substitution of tasks* between professionals. For example, nurse practitioners completed the individual care plan for all patients instead of the pediatrician. The latter reported to spend more time on more complex patients.

Third, a major facilitator for the implementation of the VAC was the fact that this eHealth innovation makes it possible for professionals to easily optimize and individualize care for the patients to *current guidelines*. The layout of the individual care plan was based on the content of the national pediatric asthma guideline, including treatment goals, medication, information, and an action plan in case of an asthma exacerbation.

#### Organizational Context

There were 3 categories related to this theme: (1) organization of care processes or organizational structure; (2) time; and (3) ICT infrastructure.

A better *organization of care* by the use of VAC was considered an important driving factor for successful implementation. For example, the easy and accessible way of communicating with patients via the VAC gave professionals the possibility to contact the patients at a time during the day that was beneficial for them. Despite some professionals experiencing *lower workload* through the use of the VAC, others emphasized the *extra workload and time* due to the extra administration for completing all information in the individual treatment plans.

Contrastingly, the involvement of the medical management at an early stage of the implementation made it easier for professionals to change work processes and, at the beginning of the implementation, spend *more time* on the use of the eHealth innovation.

A major barrier was mentioned by almost all health care professionals, namely *the lack of interoperability* (which is defined as the ability of a system to work with other systems without special effort on the part of the customer) between the VAC and current EMRs. For medicolegal reasons, professionals have to include all patients’ contacts in their personal EMR. Due to the missing link between the VAC and current EMRs, professionals had to complete both portals, which was time-consuming and not workable in daily practice. A sustainable integration is necessary to keep both systems up-to-date easily. Local ICT problems caused minor barriers, for example, firewall blocking the website.

#### Economic and Political Context

Only 1 category was defined in this theme: financial arrangements. The most important barrier for successful implementation of the VAC was the *lack of structural financial reimbursement* for Web-based monitoring. When Web-based monitoring substituted 50% of the routine outpatient visits, professionals were only reimbursed for 50% of these visits. However, they had to invest time in the use of the VAC (eg, completing the individual treatment plans, respond to messages etc) and were encouraged to reduce the number of routine outpatient visits.

## Discussion

### Principal Findings

Major perceived barriers found in this study included concerns about lack of interoperability of this innovation with other systems, the lack of structural financial reimbursement for Web-based monitoring, the burden of eHealth use on professionals’ workload, and a changing patient-professional relationship (due to less face-to-face contact).

Major perceived facilitators included training and support, a positive attitude of professionals toward eHealth, the advantages for the patients, and the possibility to tailor care to the individual patient (“personalized eHealth”), to substitute tasks between professionals, and to easily deliver care according to current guidelines by using the VAC.

We identified these barriers and facilitators for the implementation of a Web-based portal to monitor asthmatic children as a substitution for routine outpatient visits among a group of health care professionals, patients, and parents in 14 hospitals. These barriers and facilitators were arranged in 6 themes as described by Grol and Wensing: the innovation itself, individual professionals, patients, social context, organizational context, and political and economic context [20]. Although barriers and facilitators differ in scope, context, and strength, most of them were shared by professionals from different hospitals and with different functions. Findings of this study suggest and confirm that issues around implementation are multilevel and complex, and it is important to mention that no single factor was identified as a key barrier or facilitator.

The *lack of interoperability* is perhaps one of the greatest organizational obstacles to the long-term integration of eHealth infrastructure in health care [16,22,23]. As with most of the existing eHealth networks, the VAC tends to rely on custom-built systems made for specific users operating in specific settings, has a lack of open connectivity with other systems (eg, EMRs), and no information is exchanged automatically. This results in higher than necessary workload for professionals since the EMR has to contain the latest and relevant medical information due to medicolegal reasons. An adequate interface with other ICT systems is essential for acceptance, use, successful implementation, and long-term sustainability of the VAC.

The other significant obstacle for further diffusion of our eHealth innovation is the *lack of structural financial reimbursement* of Web-based monitoring [14,24]. To date, there is still a lack of payment for electronically delivered care, and funding of the current health system is solely based on reimbursement of face-to-face contacts. Using the VAC as a (partial) substitution for outpatient visits lead to production loss for hospitals and less reimbursement of these outpatient visits. Nevertheless, instead of seeing patients at the outpatient clinic, professionals now have to invest time in using the VAC (eg, completing the individual treatment plans, respond to messages, etc) for the Web-based monitoring of patients. This type of care was not yet reimbursed, and the incentive for professionals to use the VAC was solely based on their goodwill and grants. Logically, this is not sustainable and adequate funding for Web-based monitoring as a substitution for routine care, in combination with supportive policy and political vision toward eHealth, is crucial to acheiving long-term goals.

eHealth innovations have to fit the workflow of professionals to minimize *the burden of eHealth use*. Otherwise, they experience an extra workload and a time constraint, which are major impediments to implementation [14,16,25,26]. The reason for an extra workload with the introduction of the VAC consisted mostly of a lack of time to learn how to use the VAC, the work involved in transferring information between 2 systems, and an alteration in established professional roles, responsibilities, and work styles. Providing a transition period in which users become familiar with and learn how to use the eHealth innovation has been desirable to facilitate implementation [16,27].

### Limitations and Strengths

The main methodological limitation of this study is that the data were collected using 3 consecutive questionnaires over a period of 6 months. In-depth interviews to assess more detailed information about the barriers and facilitators perceived by professionals were not used. Further, we had developed a questionnaire based on the model of Grol and Wensing, which was not validated and tested for reliability. However, no valid questionnaire to assess barriers and facilitators for implementation has been published or validated. In addition, we were not able to specify similarities and differences between respondents and nonrespondents as we did not have any data of the latter. Therefore, we cannot rule out some bias. Nevertheless, we consider the response rate (51/76, 67%) as quite acceptable and therefore assume that a bias may be minimal.

For the collection of parents’ and patients’ data, we were dependent on health care professionals sending this questionnaire via the VAC because we were not allowed to have access to patients’ data. This may have influenced the response rate of this questionnaire, as it is possible that professionals did not send this questionnaire or sent it too late. Also, it would be interesting to compare the responses of the health care professionals and patients or parents in future research since their perceptions or viewpoints may be different. Another limitation could be the fact that all 3 questionnaires for professionals were sent in a time period of 6 months after starting to use the VAC. This means that patients monitored over the Web using the VAC were only seen once during this period. This could make it difficult for professionals to develop an objectively based opinion about the importance of the VAC for their daily practice. We could not objectively assess possible changes in time, differences between hospitals, or type of professionals. Also, it is possible that the time period of the study (6 months) was too short to identify all key barriers and facilitators. Nevertheless, most findings were in line with earlier studies.

An overall strength of the VAC is the involvement of health care professionals and patients from the beginning of the project. Participants’ wishes and possible objections against the VAC were used for its development, and after the RCT their feedback was used to update the VAC to optimize their use before wider implementation. Ongoing involvement of key stakeholders (especially end-users) in the design and development of eHealth technologies helps to ensure that systems are likely to be valued and used by professionals (intrinsic motivation) and should be considered as a way of overcoming barriers and facilitating implementation [16,28-31].

**Recommendations for successful implementation of an eHealth innovation.**

- Quality of care (eg, efficacy, superiority) is a major facilitator for the implementation of an eHealth innovation and appears to be relevant for the adoption of eHealth. Therefore, this should be assessed before implementation of the innovation in daily practice.
- Besides health care professionals and patients, other stakeholders (eg, information and communication technology services, governments, insurance providers) should be included as early as possible in different development and implementation phases.
- Involve end-users in the design and development of an eHealth innovation to make them develop feelings of ownership toward the innovation and to achieve a positive perception of the innovation’s usefulness and user-friendliness. Use their feedback to optimize the innovation.
- Maximize the use of and adherence to eHealth applications by developing and modifying the eHealth app from the patient’s point of view: only when a clear advantage or incentive is perceived, eHealth may sustain. The lower frequency of outpatient clinic visits is probably a major incentive in the case of the virtual asthma clinic.
- Provide adequate training and support (eg, technical assistance, training on-the-job and user manuals) during and after the implementation process to optimize use of the innovation.
- Aim for a transition period for users. This period can be used to become familiar with and learn how to use the eHealth innovation and thereby facilitate the implementation process.
- Even the best eHealth system will fail if users do not have the right knowledge and skills to use it effectively.
- Standards for technology should be optimized before or during the implementation process (interoperability, security, and privacy of hospital information and communication technology systems).
- Sufficient financial reimbursement needs to be arranged to support sustainable implementation. Chances of finding reimbursement increase with evidence of efficacy, superiority, and cost-effectiveness.
- Guarantee the privacy and safety of patients’ data.
- Implementing eHealth and sustaining it are very different ventures. After implementation, ongoing monitoring, evaluation, and adoption of the system are needed to ensure intended goals are being met and to identify ongoing barriers.

Another strength of this implementation study is that the questionnaires were based on factors identified by qualitative research, the model of Grol and Wensing [20], and thereby represent the complete spectrum of important factors related to acceptance and diffusion of an eHealth innovation. Another strong point is the fact that we obtained a representative sample of participants. Professionals from different hospitals, both secondary and tertiary care, participated in the study. Hospitals were located in both rural and urban areas. Further, professionals had different functions in the asthma team (eg, pediatric pulmonologist, pediatricians, nurse practitioners, etc). This gives a good reflection of pediatric asthma care.

Based on the barriers and facilitators found in this study, we propose a list of recommendations when implementing eHealth innovations (Textbox 1). These can be used to tailor organizational improvements to the needs of individuals and organizations.

Therefore, the suitability of the innovation in its context is essential for an innovation to become successful. Professionals can be seen and approached as key holders to achieve change and successful implementation of the innovation [7]. However, management support is essential to encourage professionals to hold on to their innovative behavior, to embrace new strategies for improving health care, to ensure sustainable use of the innovation, and to implement renewal. As mentioned, patients must also be involved in the innovation as cocreators to make them develop feelings of ownership toward the innovation and enhance positive perception of the innovation’s usefulness and user-friendliness. Only when a clear advantage or incentive is perceived by patients, are they inclined to continue to adhere to and use eHealth and, thus, it may sustain. The lower frequency of outpatient clinic visits is probably a major incentive in the case of the VAC. Besides an organizational change within the hospital, other structural changes are also necessary to achieve successful implementation of eHealth. Other stakeholders, such as the Government, policy makers, and insurance companies, have a crucial role in the implementation of eHealth, nowadays and in the future. Without adequate financial reimbursement, the introduction of innovations in health care has been doomed to fail. Sustainable solutions to finance new strategies are necessary to ensure large-scale innovations to conquer a solid place within the current health care system. At an early stage, all stakeholders together have to assess which innovations have the potential to change health care positively, from the perspective of value-based health care, efficiency, and cost savings.

### Conclusion

Implementation of an eHealth innovation is a complex, dynamic process influenced by multiple factors at the levels of the innovation itself, individual professionals, patients, and the social, organizational, and economic and political context. Understanding and defining the barriers and facilitators that influence the context appears to be important for the successful implementation and sustainability of an innovation.

Despite serious concerns about structural financial reimbursement and the lack of interoperability of the system, health care professionals, patients, and parents generally expressed enthusiasm about the VAC in our study. In the majority of the participating hospitals, this innovation became part of regular asthma care for children and is it used as a (partial) substitution of traditional outpatient care for children with asthma.

## Appendix

Multimedia Appendix 1 Barriers and facilitators for change at different levels of health care according to Model of Grol and Wensing.

Multimedia Appendix 2 Private section of the virtual asthma clinic.

Multimedia Appendix 3 Questionnaire for health care professionals.

Multimedia Appendix 4 Questionnaire for patients and parents.

Multimedia Appendix 5 Characteristics of participating hospitals.

## Abbreviations

C-ACT: Childhood Asthma Control Test
eHealth: electronic health
EMR: electronic medical record
ICT: information and communication technology
RCT: randomized controlled trial
VAC: virtual asthma clinic
