# Supplementary material for: Barriers and Facilitators When Implementing Web-Based Disease Monitoring and Management as a Substitution for Regular Outpatient Care in Pediatric Asthma: Qualitative Survey Study
Source: J Med Internet Res. 2018 Oct 30;20(10):e284. doi: 10.2196/jmir.9245 (PMC6239865; doi:10.2196/jmir.9245)
Supplement: Multimedia Appendix 1 [file jmir_v20i10e284_app1.pdf]

Multimedia appendix 1

Barriers and facilitators for change at different levels of health care according to the Model of Grol and Wensing.

**Level**	**Barriers/facilitators**
Innovation	Advantages in practice, feasibility, credibility, accessibility, attractiveness
Individual professional	Awareness, knowledge, attitude, motivation to change, behavioral routines
Patient	Knowledge, skills, attitude, compliance
Social context	Opinion of colleagues, culture of the network, collaboration, leadership
Organizational context	Organization of care processes, staff, capacities, resources, structures
Economic and political context	Financial arrangements, regulations, policies
