# Supplementary material for: Barriers and Facilitators When Implementing Web-Based Disease Monitoring and Management as a Substitution for Regular Outpatient Care in Pediatric Asthma: Qualitative Survey Study
Source: J Med Internet Res. 2018 Oct 30;20(10):e284. doi: 10.2196/jmir.9245 (PMC6239865; doi:10.2196/jmir.9245)

Communication Module

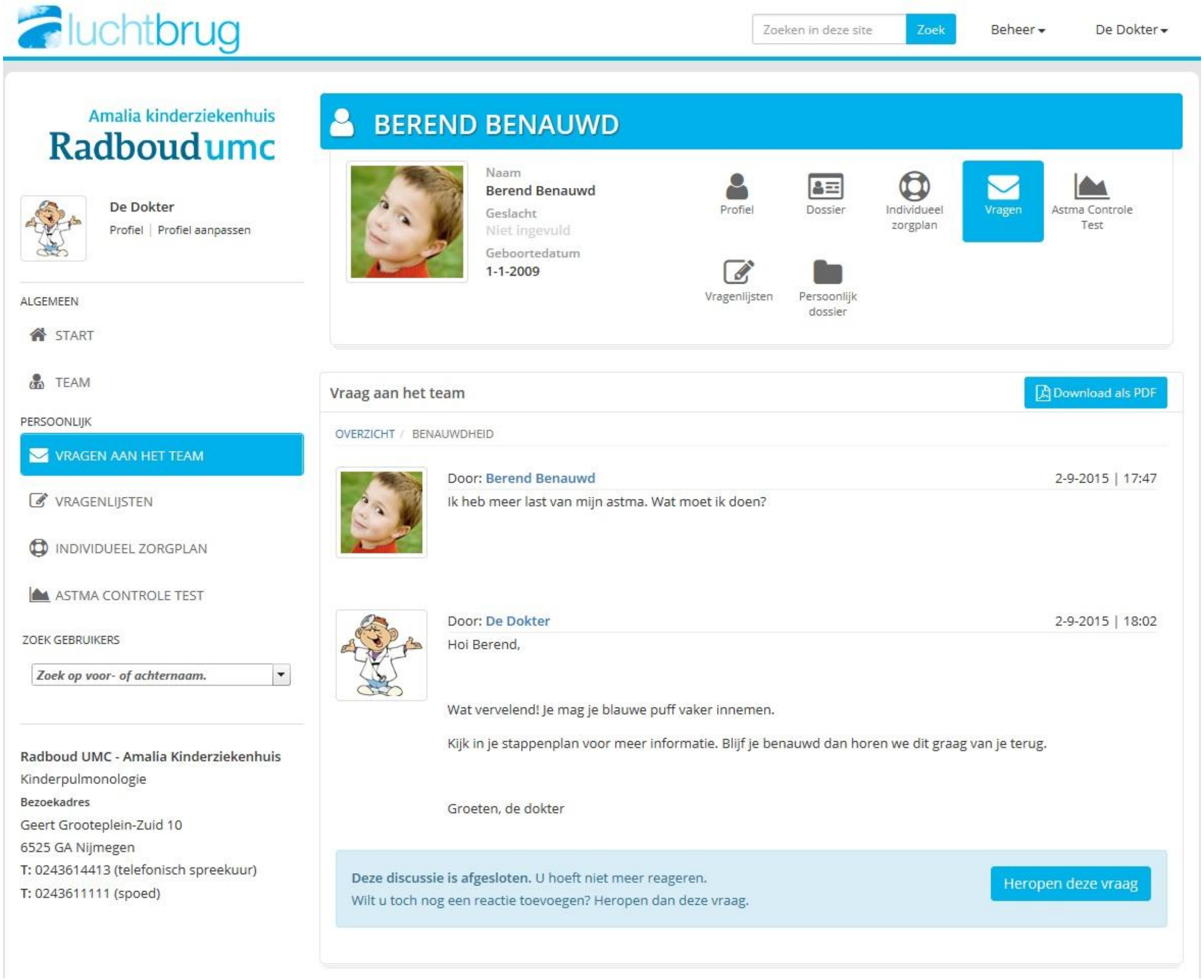

Individual treatment plan - goals

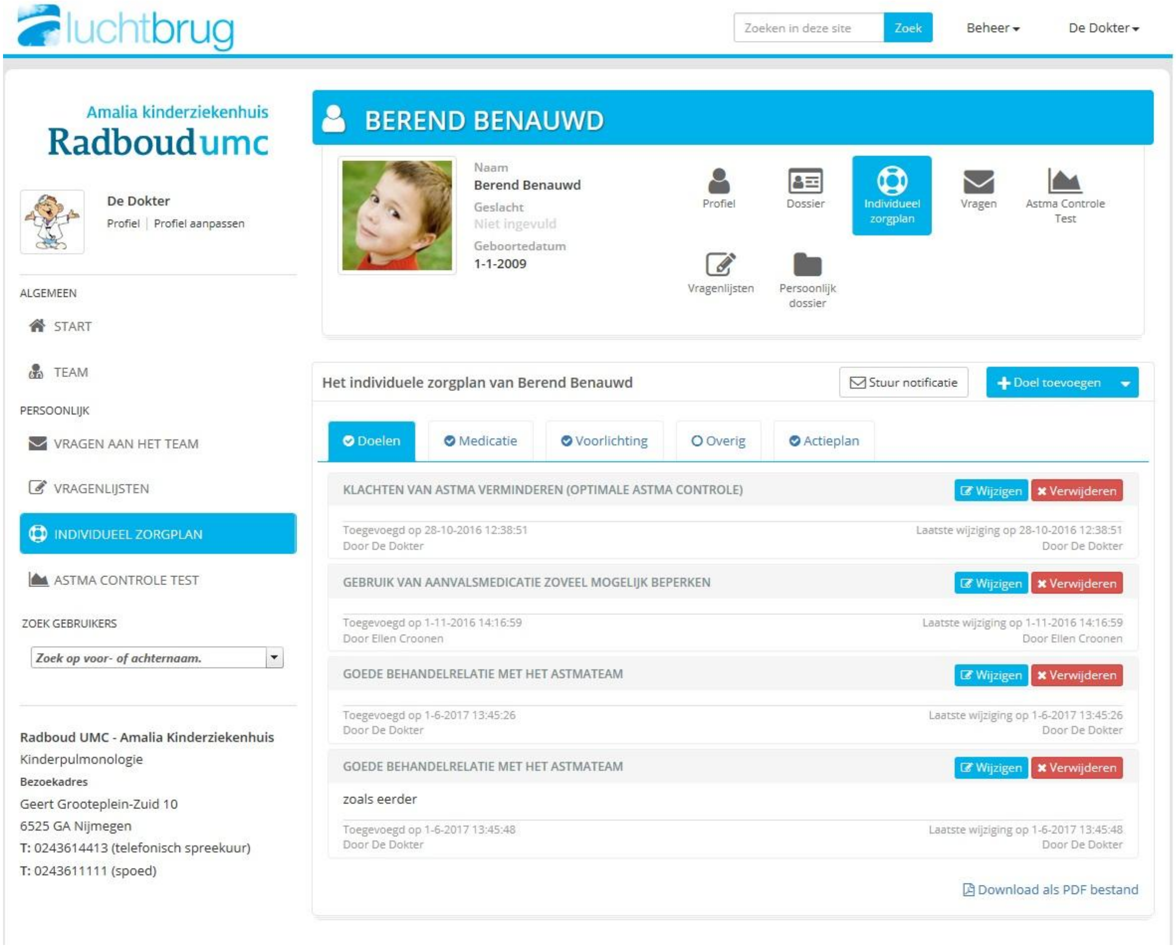

Individual treatment plan – medication

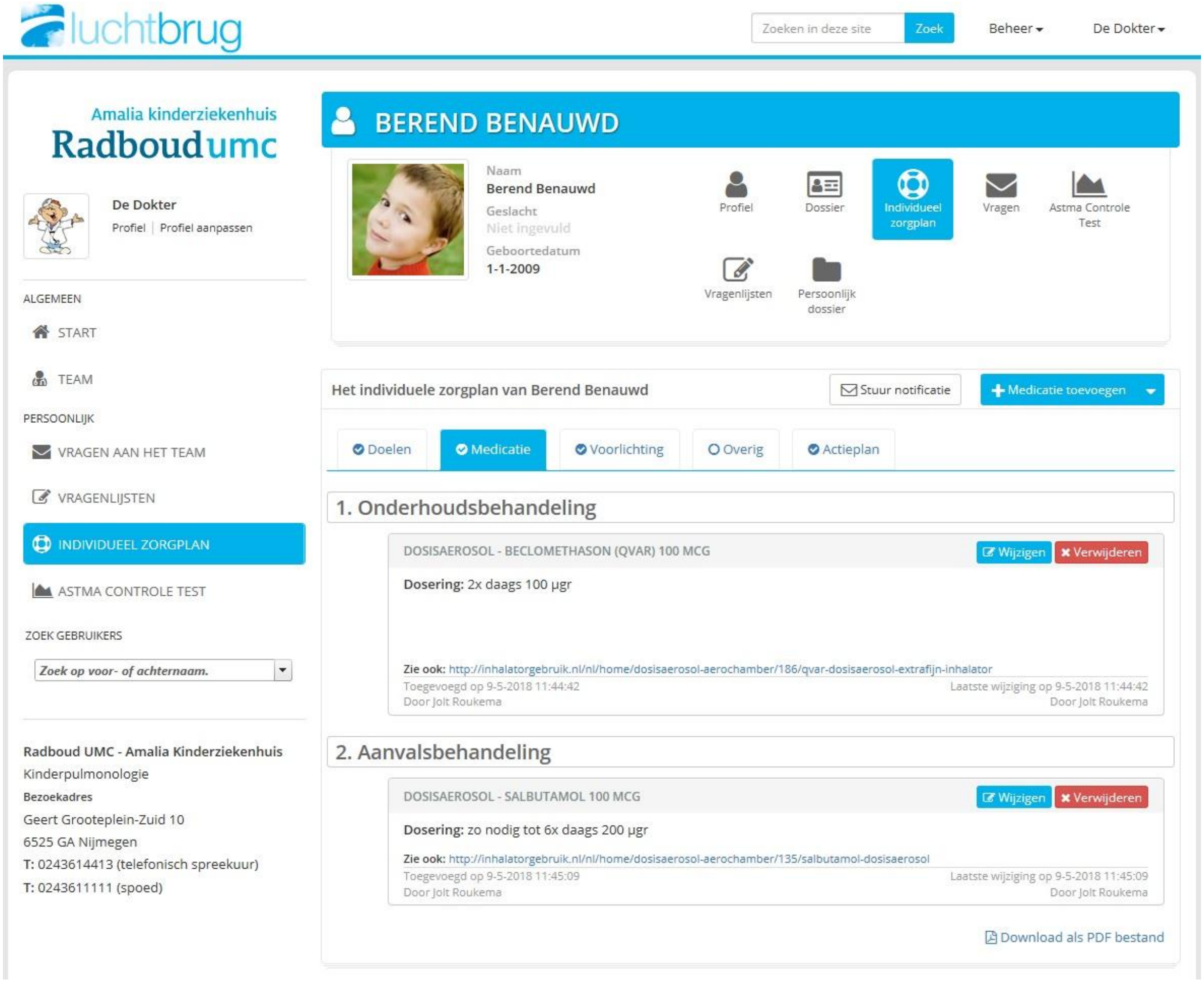

Individual treatment plan – Information and advices

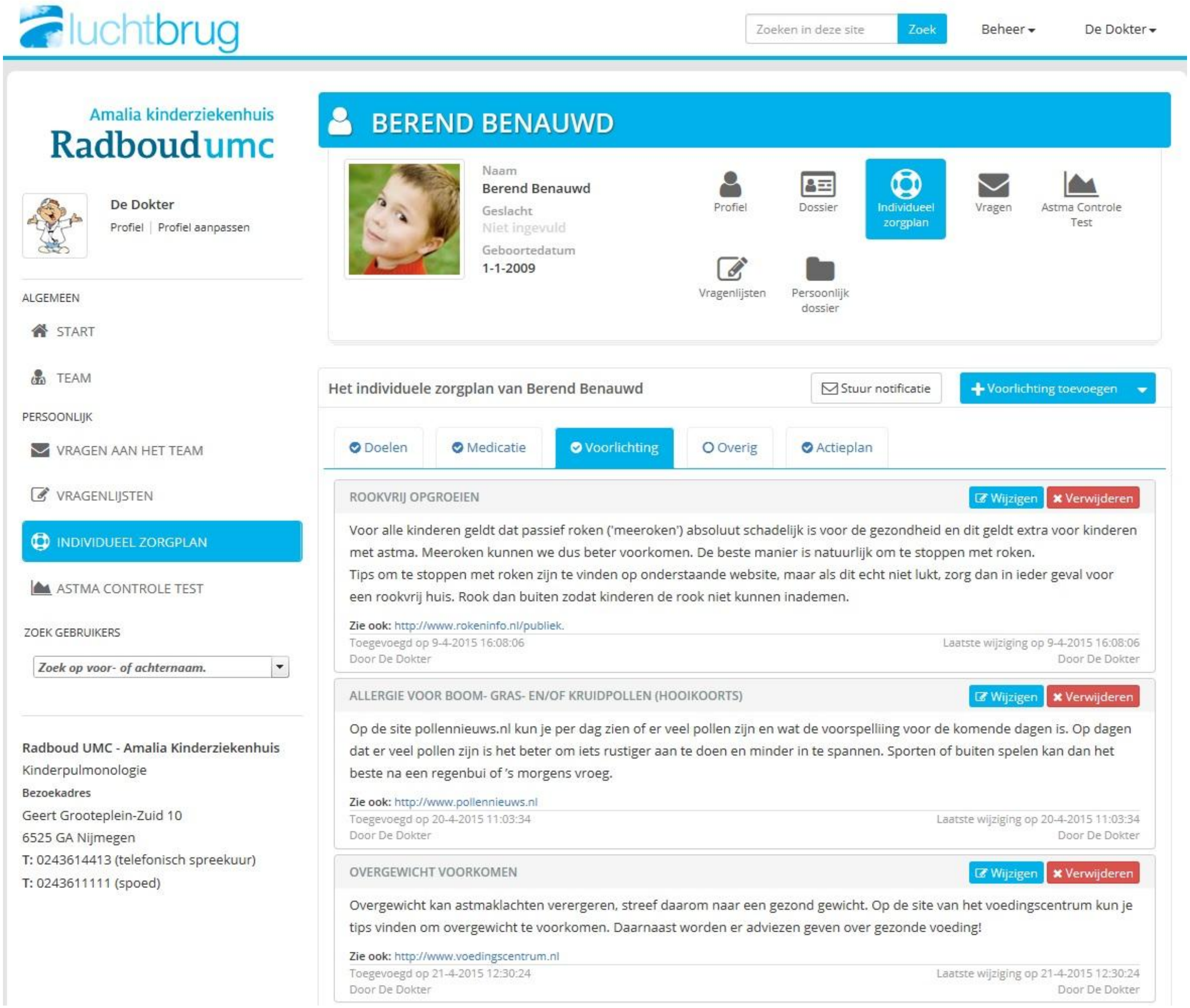

Individual treatment plan – Action plan

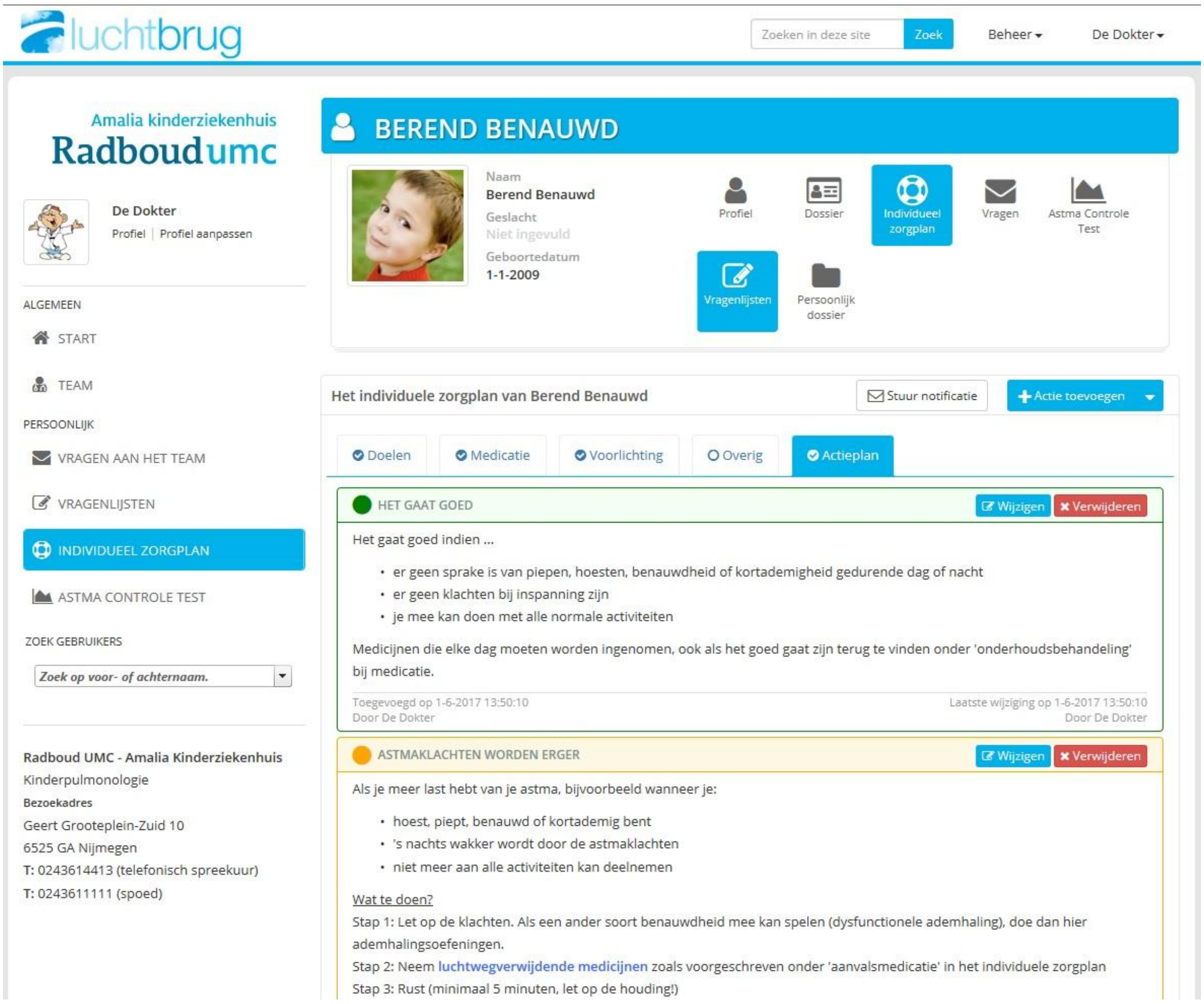

Asthma Control Test

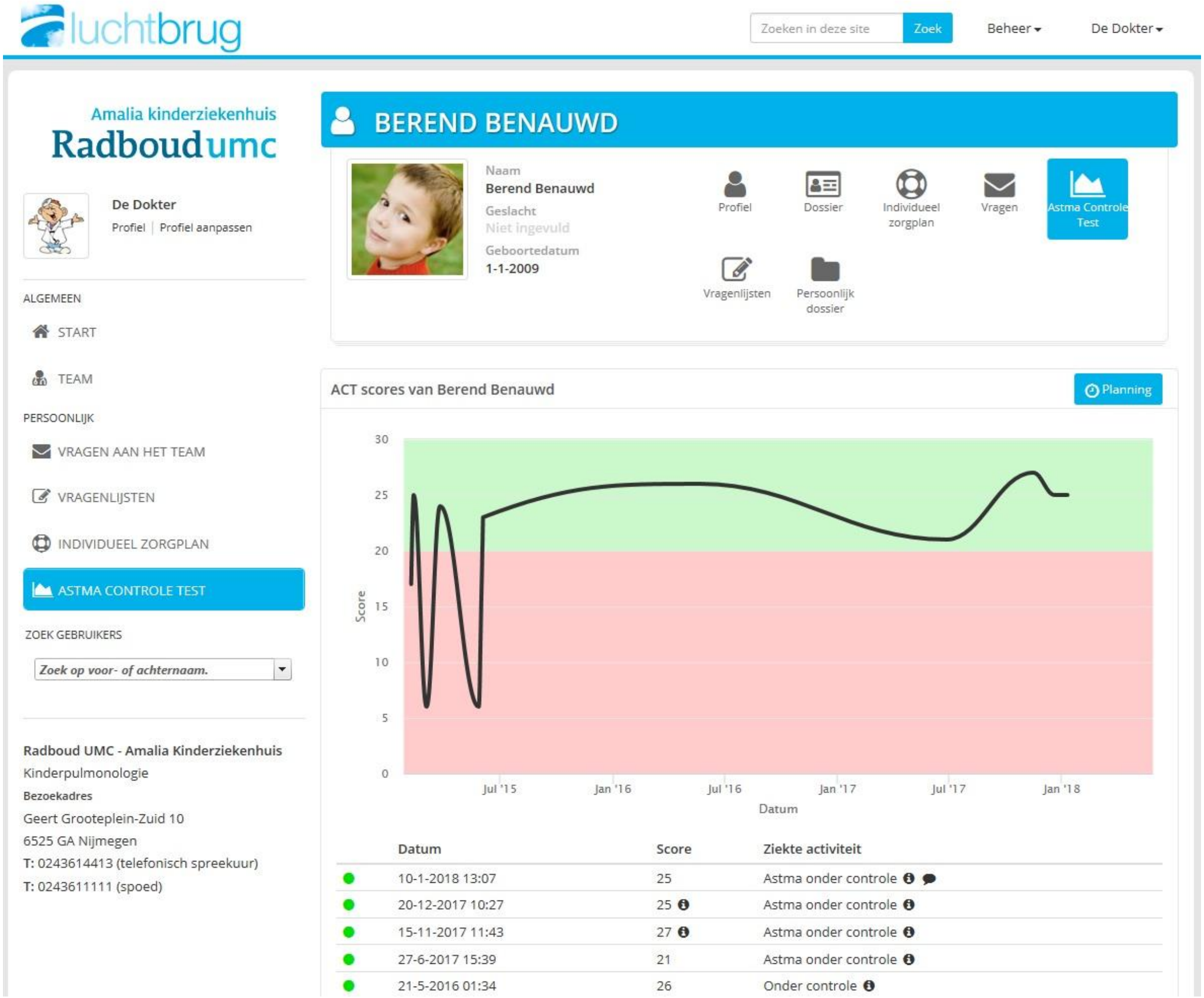

Supplement: Multimedia Appendix 2 [file jmir_v20i10e284_app2.pdf]
