# Supplementary material for: Barriers and Facilitators When Implementing Web-Based Disease Monitoring and Management as a Substitution for Regular Outpatient Care in Pediatric Asthma: Qualitative Survey Study
Source: J Med Internet Res. 2018 Oct 30;20(10):e284. doi: 10.2196/jmir.9245 (PMC6239865; doi:10.2196/jmir.9245)
Supplement: Multimedia Appendix 4 [file jmir_v20i10e284_app4.pdf]

Multimedia appendix 3

Questionnaire for patients and/or their parents

The questionnaire consisted of both open and multiple choice questions. For the multiple choice questions, a 5 point Likert scale (strongly agree, agree, neither agree or disagree, disagree and strongly disagree) was used to answer the questions.

General questions

1. What is your gender?
2. What is your age?
3. In which hospital do you go to?

Questions about the innovation

1. The VAC looks nice
2. I could easily find my way in the VAC
3. The information about asthma provided was easy to understand
4. The VAC is innovative
5. The VAC is a good addition in daily care for children with asthma

Questions about the ACT

1. De ACT questionnaires were easy to understand
2. Completing the ACT took a lot of time
3. Frequent completion of the ACT makes me more aware of my asthma
4. Frequent completion of the ACT results in a better asthma control
5. What is your role in the VAC?
6. Do you have any experience with working with an eHealth innovation?
7. Did you attend the instruction about the use of the VAC?
8. If no, in what way were you informed about the VAC?
9. Instruction about the use of the VAC was clear.
10. The length of time of this instruction was good.
11. The user manual was clearly described.

12. I have read the user manual thoroughly.
13. The distinction between the role of administrator and employee is clear to me.
14. The layout of the VAC makes it convenient for use.
15. I am sufficiently informed by my management/ department about the VAC.
16. Do you have any questions or comments about using the VAC?

<u>Questions about perceived facilitators and barriers: individual factors</u>

1. I have enough knowledge about the VAC and for who it is intended.
2. I know what they expect from me regarding to the use of the VAC.
3. I have my doubts about the VAC.
4. I am aware of the effectiveness of the VAC.
5. I have enough skills to work with the VAC.
6. I expect working with the VAC will cost me too much time.
7. I have my doubts about the information security.
8. The VAC is a good addition to the current daily pediatric asthma care.
9. I having trouble changing my (daily) routine.
10. My relationship with the patients (and/or their parents) will change due to the VAC.
11. I have my doubts about privacy regulations.
12. The VAC is user friendly
13. What is your attitude towards the use of the VAC in current pediatric asthma care?
14. What does the VAC add to the treatment of the child with asthma?
15. What is your attitude towards the use of the VAC and the online monitoring of asthma symptoms?
16. What is your opinion about the possibility for parents to communicate via the VAC?
17. How do you see the use of the VAC in addition to current electronic patients dossiers?
18. How do you think parents are opposed to the use of the VAC?
19. What are the most important reasons for your department to use the VAC?
20. Do you have any further questions or comments?

Questions about perceived facilitators and barriers: social factors

1. The use of the VAC has lead to changes within the asthma team.
2. The management has paid sufficient attention to the changing work processes due to the VAC.
3. In case of problems with the VAC I know where to go and ask my questions.
4. Parents using the VAC know who to contact in case of problems.
5. The VAC is difficult for patients with a different cultural background.
6. The VAC is difficult for patients with a lower socio-economic status.
7. Parents know how to use the VAC.

Questions about perceived facilitators and barriers: organizational factors

1. In advance, my department has set clear goals regarding the VAC.
2. The management provided adequate support for the use of the VAC.
3. The management has devoted sufficient attention to the preconditions (e.g. ICT, sustenance).
4. Everyone in the asthma team knows what their responsibilities are regarding to the use of the VAC.
5. My colleagues cooperate in using the VAC in daily practice.
6. Were there any problems at the startup of the VAC?
7. Which problems did you experience?
8. How did you solve them?
9. Are there still any problems?
10. Which problems doe you still experience?
11. How do you intend to solve these problems?
12. Have things gone differently than expected?

Questions about the (dis)advantages of the VAC

1. Do you think that the VAC, or a similar eHealth innovation, will be part of your daily practice?
2. What do you think are the advantages of the VAC for the child with asthma and/or their parents?

3. What do you think are the disadvantages of the VAC for the child with asthma and/or their parents?
4. What are the advantages of the VAC for you as a health care professional?
5. What are the disadvantages of the VAC for you as a health care professional?
6. What are the advantages of the VAC for the asthma team?
7. What are the disadvantages of the VAC for the asthma team?
8. What are the advantages of the VAC for your department?
9. What are the disadvantages of the VAC for your department?
10. Do you have any further questions or comments?

Questions about changes in the organizational processes over the last three months

1. Have there been changes over the last three months regarding to the size of the asthma team?
2. Have there been changes over the last three months regarding to the formation of the asthma team?
3. Have there been changes over the last three months regarding to the contact person for patients and/or their parents?
4. Have there been changes over the last three months regarding to frequency of consultation between nurse and physician?
