# Supplementary material for: Barriers and Facilitators When Implementing Web-Based Disease Monitoring and Management as a Substitution for Regular Outpatient Care in Pediatric Asthma: Qualitative Survey Study
Source: J Med Internet Res. 2018 Oct 30;20(10):e284. doi: 10.2196/jmir.9245 (PMC6239865; doi:10.2196/jmir.9245)
Supplement: Multimedia Appendix 5 [file jmir_v20i10e284_app5.pdf]

Multimedia appendix 5

Characteristics of participating hospitals

**Hospital**	**Area**	**Participating health care professionals**	**Implementation**
Academic	Urban	4	Successful
Academic	Urban	3	Unsuccessful
Academic	Urban	4	Unsuccessful
General	Urban	16	Successful
General	Urban	2	Successful
General	Urban/Rural	4	Successful
General	Urban	3	Successful
General	Urban	5	Unsuccessful
General	Urban	7	Successful
General	Rural	4	Successful
General	Urban	6	Successful
General	Rural	10	Successful
General	Rural	3	Unsuccessful
General	Urban	4	Successful
